# Language Distance Moderates the Effect of a Mixed-Language Environment on New-Word Learning for 4-Year-Old Children

**DOI:** 10.3390/brainsci14050411

**Published:** 2024-04-23

**Authors:** Zhengkai Niu, Zilong Li, Yunxiao Ma, Keke Yu, Ruiming Wang

**Affiliations:** Philosophy and Social Science Laboratory of Reading and Development in Children and Adolescents (South China Normal University), Ministry of Education, & Center for Studies of Psychological Application, School of Psychology, South China Normal University, Guangzhou 510631, China; 20219647@m.scnu.edu.cn (Z.N.); 2021010238@m.scnu.edu.cn (Z.L.); 2020010230@m.scnu.edu.cn (Y.M.); kkyu@m.scnu.edu.cn (K.Y.)

**Keywords:** language mixing, vocabulary acquisition, language distance

## Abstract

As bilingual families increase, the phenomenon of language mixing among children in mixed-language environments has gradually attracted academic attention. This study aims to explore the impact of language mixing on vocabulary acquisition in bilingual children and whether language distance moderates this impact. We recruited two groups of bilingual children, Chinese–English bilinguals and Chinese–Japanese bilinguals, to learn two first-language new words in a monolingual environment and a mixed-language environment, respectively. The results showed that the participants could successfully recognize the novel words in the code-switching sentences. However, when we compared the performance of the two groups of bilingual children, we found that the gaze time proportion of the Chinese–English bilingual children under the code-switching condition was significantly higher than that of the Chinese–Japanese bilingual children, while there was no significant difference under the monolingual condition. This suggests that language mixing has an inhibitory effect on vocabulary acquisition in bilingual children and that this inhibitory effect is influenced by language distance, that is, the greater the language distance, the stronger the inhibitory effect. This study reveals the negative impact of language mixing on vocabulary acquisition in bilingual children and also implies that there may be some other influencing factors, so more research is needed on different types of bilingual children.

## 1. Introduction

With the development of globalization, more and more people need to use and communicate in multiple languages, which forms a mixed-language environment. A mixed-language environment refers to the interaction and influence between languages in the context of bilinguals using two or more languages. Similarly, a mixed-language environment is a common linguistic phenomenon, which not only reflects the linguistic diversity and plurality of people but also affects their language learning and use [1]. This article focuses on children, a special language-learning group, who need to acquire and use two or more languages simultaneously in a mixed-language environment, which has a significant impact on their language and cognitive abilities.

### 1.1. Vocabulary Learning in Children in a Mixed-Language Environment

Language mixing refers to the process of different linguistic components of two or more languages influencing and combining with each other at a certain social or individual level, forming new linguistic forms or systems [2,3]. There are two types of language mixing: code-mixing and code-switching. Code-mixing is a situation in which people use another language when people mix two (or more) languages or variations in a speech act or discourse without anything in the language situation demanding the mixing of the language [4,5,6]. If speech-mixing or a combination of different variations occurs in the same clause, then the occurrence is called code-mixing.

Code-switching, as a linguistic phenomenon, is complex and, as such, has been an area of research over many years. With regard to defining the phenomenon of code-switching, different definitions have been provided by linguists. One of these definitions sees code-switching as the alternation of different languages or dialects within a single language act or between different language acts [7]. According to Muysken [8] code-switching refers to any instances in which a phrase contains vocabulary or grammatical elements from two different languages. Cook [9] defines code-switching as a phenomenon in which proficient bilinguals/multilinguals, when both speakers speak the same two languages, switch between them mid-sentence. Bullock & Toribio [10] state that code-switching refers to the capacity of using, replacing, or switching two languages in use. In this case, the speaker at least has the proficiency of the second language in addition to the first language so that they might switch from the first language to the second language and vice versa. Thelander, as cited in Chaer & Agustina, states that code-switching is the use of two language (or more) by a speaker in the same communication event [11]. 

Many linguists divide code-switching into three terms: tag-switching code-switching, inter-sentential code-switching, and intra-sentential code-switching [12,13]. Tag-switching refers to code-switching that belongs to the units of independent elements contained in a statement or a question; tag-switching usually appears at the beginning or at the end of a statement or question. Inter-sentential switching is an alternation in a single discourse between two languages, where the switching occurs after a sentence in the first language has been completed, with the next sentence starting with a new language, or, on the other hand, it can mean that inter-sentential code-switching occurs between a different number of sentences and refer to a switch from one language to another in a sentence that involves syntactic units of words, phrases, or clauses. This study focuses on language mixing based on intra-sentential code-switching. 

For language acquisition, vocabulary learning abilities are also an important part. Vocabulary learning ability refers to the ability to acquire and memorize the meanings of new words from a language input, which is the foundation of language learning and also a key indicator of children’s cognitive development [14,15]. Vocabulary learning abilities are influenced by various factors, in addition to external factors such as children’s age, gender, intelligence, motivation, attention, memory, etc.; language mixing also has an inevitable and significant impact on children’s vocabulary learning abilities [16].

However, there is no unanimous conclusion on what kind of effect language mixing has on vocabulary acquisition. Previous studies have shown that language mixing has both positive and negative effects on children’s vocabulary learning abilities, which depend on factors such as children’s age, language distance, language input amount, language switching type, etc. [17]. Of course, children’s vocabulary learning abilities are also affected by the language environment they are in, especially when they are exposed to two or more languages or when they need to switch between different languages, which may have positive or negative effects on their vocabulary learning abilities [18].

Children are considered to be the best language learners, as they have higher language sensitivity and plasticity, and can unconsciously acquire language in a natural language environment [19,20,21,22]. Language mixing in bilingual children is considered to be a conscious, purposeful, and regular behavior, which reflects their language choice and adaptation abilities. Place & Hoff tagged 30 min time blocks where both languages were used (within or across speakers) as mixed blocks. The exposure to English-dominant mixed blocks was positively linked to these 30-month-old infants’ English language skills [23]. The relation remained significant after removing the effect of the English-only blocks. Moreover, within contexts involving language mixing, infants who heard more words were also the ones who produced more vocalizations [24]. Bail et al. reported a positive correlation between total vocabulary and conceptual vocabulary in both of the languages that the toddler in their study had been exposed to and the degree of language mixing of the parents during a 13 min play session [25]. Language mixing can help children to better understand and express their thoughts and emotions, thus increasing their language creativity and expressiveness [9]. At the same time, language mixing is a ubiquitous linguistic phenomenon, which is not a manifestation of language confusion or error but a conscious and purposeful language choice, which can achieve various communicative functions, such as emphasis, supplement, quotation, clarification, adjustment, intimacy, etc. [26].

However, negative effects of language mixing on vocabulary acquisition have also been found. Yang explored the representation and reasoning of periodic time by Cantonese–Mandarin bilingual children and Mandarin monolingual children and found that the time expression mode of Cantonese affected the representation and reasoning of periodic time by Cantonese–Mandarin bilingual children. Regardless of whether the time was in units of 5, not in units of 5, or in units of “words”, the response times of Cantonese and Putonghua bilingual children were longer than those of Putonghua monolingual children [27]. This shows that bilingual children have cognitive conflicts and interference when processing time information in two languages. There are two main reasons for the negative impact of language mixing, the first being that language mixing increases children’s cognitive load and interferes with their language input. Cognitive load refers to the amount of cognitive resources required to complete a task, while language processing and output refers to the mental activities completed when understanding and producing language, including phonetics, vocabulary, grammar, and pragmatics [28]. In the situation of language mixing, children need to switch between different languages, which requires them to activate and inhibit the relevant knowledge of two or more languages at the same time, thus increasing their cognitive load [29]. This increase in cognitive load may interfere with children’s language processing and output, causing them to have difficulties or errors in understanding and producing language, such as forgetting, confusing, replacing, or misusing vocabulary, irregular or inconsistent grammar, inappropriate or unsuitable pragmatics, etc. [30]. These difficulties or errors may affect children’s mastery of the vocabulary and grammar in each language, thus affecting their language accuracy and fluency. In addition to increasing cognitive load, language mixing may also reduce the amount of input each language provides to children, reducing their exposure to and practice of the language. In the growth environment of bilingual children, the input amount of each language will be reduced by about half, which may lead to the lack of mastery of the vocabulary and grammar of each language for the children [14]. The reduction in language input amount may reduce children’s language exposure and practice opportunities, thus affecting their language acquisition and development [31].

Heinlein et al. further showed that language mixing has a negative effect on children’s new vocabulary acquisition [17]. They used a novel word-learning task to examine the effect of parental mixed language use on children’s vocabulary comprehension abilities, with 3-year-old French–English bilingual children and Spanish–English bilingual children as the subjects. They found that the higher the parental mixed language use rate, the lower the children’s vocabulary comprehension ability, which indicates that a mixed-language environment has an inhibitory effect on children’s new vocabulary acquisition, and this inhibitory effect begins to appear from 1.5 years of age [17]. This result is consistent with other studies that show that bilingual children in a mixed-language environment lag behind monolingual children in language and vocabulary learning [29,32,33,34]. Fernández-Dobao and Herschensohn used two writing tasks to compare the grammatical ability of Spanish bilingual children and monolingual children. They found that bilingual children showed overregularization phenomena when using inflected verbs, indicating that bilingual children were not as good as monolingual children in learning and using Spanish verb morphology. They argued that this was because bilingual children had to master two different verb morphology systems at the same time, thus facing more linguistic complexity in language learning [32]. Morini et al. also supported this view. They used mixed-language sentences as materials to explore the vocabulary recognition ability of bilingual toddlers aged 18–24 months in Spanish–English. They found that bilingual toddlers could successfully recognize familiar words in any language environment [33]. Hammer et al. also found that bilingual children’s language and vocabulary learning level developed slower than that of monolingual children, and they needed more time to develop second-language skills [29]. From the above studies, we can see that language mixing has an inhibitory effect on the vocabulary development of bilingual children.

### 1.2. The Moderating Effect of Language Distance on the Influence of Mixed-Language Environments on Vocabulary Learning

In addition to the mixed-language environment, language distance also affects vocabulary acquisition in bilingual children. Two kinds of bilinguals with different linguistic distances are also exposed to different mixing environments, which may lead to changes in the extent and direction to which language mixing affects vocabulary acquisition. Language distance refers to the degree of difference between two languages, usually based on the comparison of phonetics, grammar, semantics, writing, etc. [35]. The similarity of two languages in terms of grammar, vocabulary, writing, pronunciation, etc., is also known as “closeness”: the closer a language is to the target language, the smaller the language distance, and vice versa [36]. The choice of languages with different language distances can affect the difficulty and frequency of language switching and mixing in bilinguals as well as the difference in the vocabulary learning abilities of bilinguals [28]. Cui used the word lists and procedures provided by the Automated Similarity Judgment Program (ASJP) database to calculate the LDND values for Chinese and 30 other languages. These values represent the vocabulary distance between two languages. Cui’s study found that the more linguistically distant the learners’ mother tongue was from the Chinese language, the less effective they were in learning Chinese [37]. Regarding the impact of language distance on bilingual learning, there are currently two different views. One view holds that the closer the language distance, the more positive the effect of language learning is for children in a mixed-language environment [37]. Muñoz compared the receptive English grammar skills of two groups of 7- and 9-year-old Danish children at the beginning of English second-language (L2) teaching and the receptive English grammar skills of two groups of Spanish/Catalan children of the same age after several years of teaching [38]. The results showed that the English grammar skills of Danish children were better than those of Spanish/Catalan children, and this difference was not caused by the time or quality of formal teaching but by the fact that the language distance between Danish and English was smaller than that between Spanish/Catalan and English, making Danish children more receptive to English grammar knowledge. This literature suggests that, for children, the closer the language distance, the more positive the effect of language learning is in a mixed-language environment. Another view holds that the closer the language distance, the more negative the effect of language learning is for children in a mixed-language environment. Ou and Liu, using Mongolian students as a case study, analyzed the cross-linguistic influence of language distance on Chinese–English translation and its underlying causes. Their research revealed that the impact of language distance surpasses that of language type, and the language distance perceived by children directly affects their acquisition of the target language, potentially facilitating or impeding language transfer [39]. In summary, language distance may moderate the effect of language learning for children in a mixed-language environment. Therefore, exploring the relationship between language distance and vocabulary learning abilities is of great significance for understanding the language performance and cognitive development of bilinguals. However, it is not clear whether bilingual children have different abilities to learn new words in mixed-language environments with different language distances, which is a question worth further exploration.

By analyzing the above research results, we found some unresolved issues. First, the existing studies did not fully consider the children’s familiarity with the vocabulary when using the language materials. For example, Heinlein et al. used pseudowords that conformed to the morphology as the target words for learning [17], while Morini et al. used words which the children had been familiar with as the target words for their study [33]. In these two situations, there was a significant difference in the children’s familiarity with the target words, which might have affected their language processing and learning outcomes. Therefore, this study used pseudowords as the language materials to avoid the interference of too-high or too-low familiarity on the children.

Second, the existing studies did not control the age group of the children when recruiting the subjects. Different studies covered children from 18 months to 9 years of age, and these children had great differences in their language level and cognitive abilities. Brooks argued that children should not receive too-complex inputs in their early language learning, because their cognitive ability has not developed enough [34]. On the contrary, children enter a rapid language development stage at around 3 years of age, when they begin to master a large vocabulary and can understand and use simple grammatical structures. According to the stages of children’s language development, 3–5 years of age is the period of rapid development of children’s oral expression, during which children can use simple sentences and more complex sentences, master most grammatical structure forms, and have a little understanding of the abstract relationships between words [40]. Therefore, this study chose 4-year-old children as the subjects, which is a suitable age group for studying new-word learning, neither too early nor too late.

Finally, the existing studies did not fully consider the impact of language distance when choosing the language types. Language distance refers to the similarity or difference between two languages.

A study on the acquisition of Dutch found that linguistic distance facilitates vocabulary acquisition [41]. However, the Chinese language is specialized and non-Chinese studies are not necessarily transferable to Chinese. For example, Chinese–English bilingual children have been shown to make different types of errors in English writing from their peers who are native English speakers [42]. In addition, a study explored linguistic distance and second-language effect on the lexical processing of Chinese, English, and Japanese trilinguals. It was found that trilinguals whose first language was Chinese showed a different second-language effect than non-native Chinese speakers; linguistic distance had a unique effect on the lexical processing of native Chinese speakers [43]. The cognitive neural mechanism of Chinese vocabulary processing may be different from that of European languages, so language distance research based on European languages as the target language and language distance research based on Chinese as the target language may have a large bias [44].

### 1.3. Hypotheses of the Present Study

Drawing on the above, the present study employed an experimental method, using 4-year-old Chinese–English and Chinese–Japanese bilingual children as the participants and sentences composed of words with a medium familiarity and novel pseudowords as the language stimuli to compare their ability and performance in learning new words in mixed-language environments with different language distances and different language switch types. To control the influence of the family’s mixed-language environment on the children, the present study selected children from the same kindergarten who enrolled at the same time and had been learning for one year as the participants. Some of the children were learning Chinese–English bilingualism, while others were learning Chinese–Japanese bilingualism. The purpose of the present study was the following: 1. to investigate whether and how a mixed-language environment affects the learning of new words by 4-year-old children; and 2. to examine whether language distance (Chinese–English and Chinese–Japanese) has a moderating effect on the ability of children to learn new words in a mixed-language environment. This study used an eye-tracking paradigm based on eye movement technology as the experimental task, which required the participants to selectively gaze at one of the two pictures appearing on a screen, based on the target word in a sentence, which is an effective method to measure children’s vocabulary learning effect [8]. The expected results of the present study were that the mixed-language environment would have an impact on the children’s ability to learn new words and that the language distance would alter the degree of this impact. The present study has important implications and value for understanding the mechanism of children’s language acquisition, promoting children’s multilingual and cross-cultural communication abilities, and providing guidance and suggestions for children’s language education and intervention.

## 2. Experiment 1: The Effect of a Mixed-Language Environment on New-Word Learning in 4-Year-Old Children

### 2.1. Materials and Methods

#### 2.1.1. Purpose

Experiment 1 used an eye-tracking paradigm to investigate whether and how a bilingual environment affected the learning of novel words by 4-year-old children.

#### 2.1.2. Participants

Experiment 1 recruited 19 Chinese–English bilingual children from a kindergarten in Lanzhou, China, among whom 3 did not meet the experimental requirements and were excluded from the group. The average age of the participants was 4.069 ± 0.239, including 12 boys and 4 girls. All the participants had been learning bilingualism for 1 year, with Chinese as their native language and American standard English as their second language. The recruitment criteria for this experiment were the following: 1. The participants had to be 4 years old. 2. The participants’ families did not have a bilingual environment, and the most frequently heard language was Chinese according to parental reports. 3. The participants came from families with the same socioeconomic status (SES). SES is a complex and multidimensional construct, including independent objective characteristics (such as income or education) and people’s subjective evaluations of their position in the socioeconomic spectrum [45]. Since the research participants were children who did not have independent occupations or incomes and their bilingual environment was a full-time kindergarten, this meant that they were in the same mixed-language environment during their one-year bilingual learning process. Therefore, we did not need to use the SES questionnaire to measure the socioeconomic status of these children [46,47,48]. 4. They had enrolled in the same time period and had no transfer experience. 5. All the participants had no neurological history, typical hearing, vision, or corrected vision. They all participated in the experiment accompanied by their parents and teachers, had informed consent forms signed on their behalf before the start of this study, and received compensation after this study was completed. This study was approved by the Ethics Review Committee of South China Normal University.

#### 2.1.3. Design

Experiment 1 adopted a single-factor two-level (learning context: monolingual, bilingual) within-subject design. In this study, the selection of target words as well as the indicators of the vocabulary acquisition effects referred to Byers’ study [8].

#### 2.1.4. Materials

Experiment 1 consisted of 24 materials, each composed of auditory and visual stimuli. There were 12 materials for each of the learning and testing phases. The auditory stimuli included 24 sentence materials, 12 of which were monolingual sentences, used in the learning phase, and 12 were mixed-language sentences, used in the testing phase. Each sentence contained a familiar word and a target word. The familiar words were one of three animals (cat, dog, pig). The participants were familiar with the three animals and knew and recognized their spelling and pronunciation. The target words were one of two novel words (“艾库 (Aiku)”, “克莫 (Kemo)”). The pronunciation of both target words was bisyllabic, and their pronunciation did correspond to that of any fixed object with the same or a similar pronunciation. The two target words corresponded to two novel objects that did not exist in the real world. The novel objects were three-dimensional objects made by 3DMAX (Adobe.hisua.cn), and geometric modifications were applied to them to make them less similar to any known objects. The correspondence between the two target words and the two objects was consistent within subjects (e.g., for a certain participant, 艾库 (Aiku) corresponded to the more rounded one and 克莫 (Kemo) to the more irregular one).

The monolingual sentences were entirely in Chinese, with familiar words (animal words) and target words (pseudowords), such as “你能看到猫在艾库上面吗? (Can you see the cat on the Aiku?)”. The mixed-language sentences were mostly the same as the monolingual sentences, except for the fact that the familiar words were replaced with English expressions, such as “你能看到cat在艾库上面吗? (Can you see the cat on the Aiku?)”.

The target words were balanced across sentences, and the sentence types were balanced for the participants. The visual stimuli also included 24 picture materials, 12 of which were pictures of novel objects (艾库 (Aiku), 克莫 (Kemo)) combined with familiar animals (cat, dog, pig), used in the learning phase, and 12 were pictures of only novel objects (艾库 (Aiku), 克莫 (Aiku)), used in the testing phase. The novel objects were balanced across the pictures, and the picture types were balanced for the participants.

#### 2.1.5. Procedure

The experiment consisted of two phases: a learning phase and a testing phase. 

During the learning phase, the participants were seated in a quiet room, in front of a 24-inch MSI computer monitor with a Tobii Eye Tracker 5 (Tobii Technology, Inc., Reston, VA, USA) installed on it. The screen was adjusted according to the participants’ height to be parallel to their line of sight, to ensure that they could clearly see the content on the screen. Each time a stimulus was presented, the visual stimulus picture and the auditory stimulus sentence appeared simultaneously. The screen was divided into two, and two different pictures appeared on both sides of the screen, with the position of the pictures balanced in the experiment. The participants were instructed to pay attention to the pictures on the screen and listen to the guidance of the sentences. After each experimental trial, a blank screen was provided to attract the children’s gaze to recalibrate the eye movement. 

**Figure 1:**
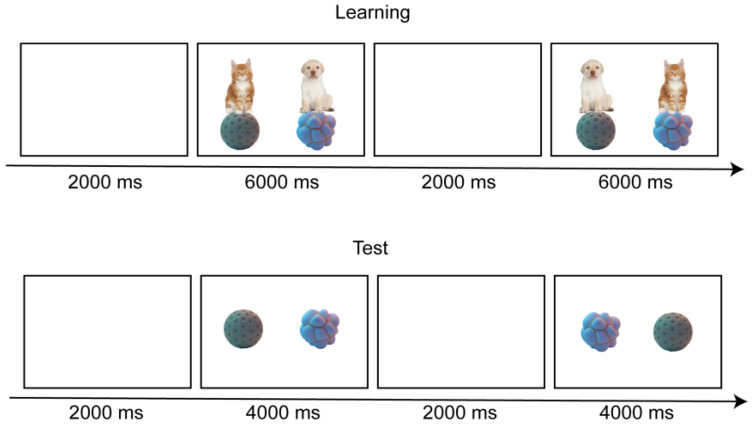
Flowchart of Experiment 1.

The specific procedure of the learning phase was as follows: First, a blank screen was presented, and, after 2000 milliseconds, the auditory and visual materials appeared together, and the auditory stimuli guided the children to observe the familiar target in either a monolingual or mixed-language sentence. For example, with the dog and a more rounded object below it on the left side of the screen and the cat and a more irregular object on the right side of the screen, the sentence played in the monolingual context was “你能看到猫在艾库上面吗? (Can you see the cat on the Aiku?)”. In the mixed-language context, the same picture was played as “你能看到cat在艾库上面吗? (Can you see the cat on the Aiku?)”. The auditory stimuli lasted 4000 milliseconds, and the visual stimuli lasted 6000 milliseconds, with a total duration of 8000 milliseconds for each learning experiment. Then, the next trial began. The specific procedure of the testing phase was as follows: First, a blank screen was presented, and, after 2000 milliseconds, the auditory and visual materials appeared together, and the auditory stimuli guided the children to pay attention to the target object. The auditory stimuli lasted 2000 milliseconds, and the visual stimuli lasted 4000 milliseconds. Each testing trial lasted 6000 milliseconds. Then, the next trial began (Figure 1). 

The experimenter monitored the experimental status and recorded the fixation time through the built-in camera of the eye tracker and used Python to set up and control the experiment. The total duration of the experiment was about 4 min.

### 2.2. Results

**Figure 2:**
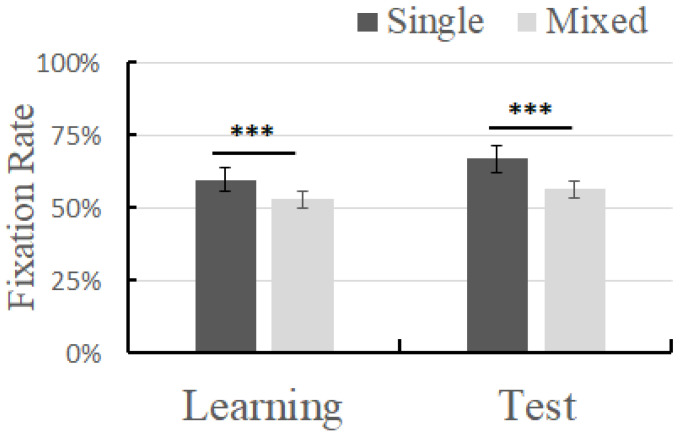
Fixation time of different learning contexts in different phases; error bar represents the standard deviation. *** *p* < 0.001.

First, a one-sample *t*-test was performed on all the trials of the four conditions to ensure that the children’s learning effect was higher than the random level. The results showed that the target fixation time ratio in the mixed-language context in the learning phase was significantly higher than 50% (*t*(155) = 11.94, *p* < 0.001) (Table 1); the target fixation time ratio in the monolingual context in the learning phase was significantly higher than 50% (*t*(155) = 23.40, *p* < 0.001); the ratio in the mixed-language context in the testing phase was significantly higher than 50% (*t*(25) = 11.07, *p* < 0.001); and the ratio in the monolingual context in the testing phase was significantly higher than 50% (*t*(25) = 17.98, *p* < 0.001). A paired-sample *t*-test was performed on the average fixation time of the target words for each participant under different learning contexts (monolingual, mixed-language). The results showed that, in the monolingual context, in the learning phase, the participants’ fixation time on the target was significantly higher than in the mixed-language context (*t*(12) = 11.881, *p* < 0.001, *Cohen d* = 3.925) In the testing phase, in the monolingual context, the participants’ fixation time on the target was significantly higher than in the mixed-language context (*t*(12) = 7.562, *p* < 0.001, *Cohen d* = 1.504) (Figure 2).

## 3. Experiment 2: The Effect of Language Distance on the Effectiveness of Vocabulary Acquisition

### 3.1. Materials and Methods

#### 3.1.1. Purpose

The purpose of Experiment 2 was to explore whether language distance would modulate the ability of children to learn new words in a mixed-language environment.

#### 3.1.2. Participants

Experiment 2 recruited 17 Chinese–Japanese bilingual children from a kindergarten in Lanzhou, China, among whom 4 did not meet the experimental requirements and were excluded from the group. The average age of the participants was 4.057 ± 0.251, including 11 boys and 6 girls. All the participants had been learning bilingualism for 1 year, with Chinese as their native language and standard Japanese as their second language. The recruitment criteria for Experiment 2 were the same as in Experiment 1.

#### 3.1.3. Design

Experiment 2 adopted a single-factor two-level (learning context: monolingual, mixed-language) within-subject design.

#### 3.1.4. Materials

The experimental materials used in Experiment 2 were the same as those in Experiment 1. The monolingual sentences were identical to those in Experiment 1. The monolingual sentences were entirely in Chinese, with familiar words (animal words) and target words (pseudowords), such as “你能看到艾库上面的猫吗? (Can you see the dog on the Aiku?)”. The target word in the mixed-language sentence was English in Experiment 1 and changed to Japanese in Experiment 2. The mixed-language sentences were mostly the same as the monolingual sentences, except that the familiar words were replaced with Japanese expressions, such as “你能看到艾库上面的ねこ吗? (Can you see the cat on the Aiku?)”.

#### 3.1.5. Procedure

The procedure was the same as that in Experiment 1.

### 3.2. Results

**Figure 3:**
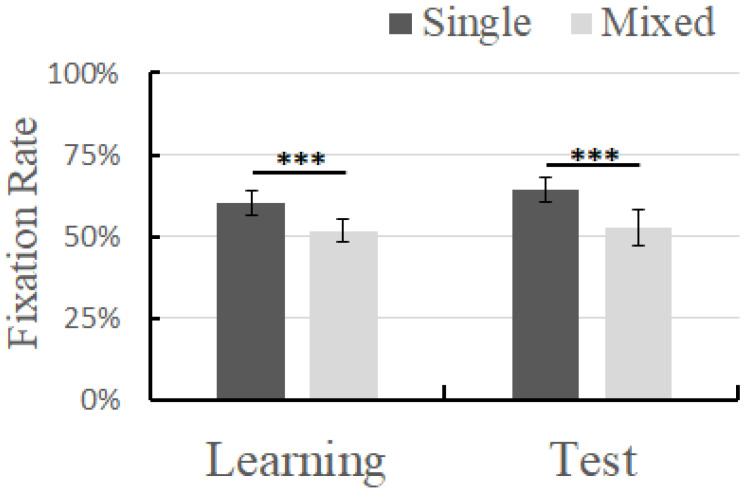
Fixation time of different learning contexts in different phases; error bar represents the standard deviation. *** *p* < 0.001.

First, a one-sample *t*-test was performed on all the trials of the four conditions to ensure that the children’s learning effect was higher than the random level. The results showed that the target fixation time ratio in the mixed-language context in the learning phase was significantly higher than 50% (*t*(83) = 4.25, *p* < 0.001) (Table 2); the target fixation time ratio in the monolingual context in the learning phase was significantly higher than 50% (*t*(83) = 25.84, *p* < 0.001); the ratio in the mixed-language context in the testing phase was significantly higher than 50% (*t*(13) = 1.83, *p* < 0.05); and the ratio in the monolingual context in the testing phase was significantly higher than 50% (*t*(13) = 13.73, *p* < 0.001). A paired-sample *t*-test was performed on the fixation time of the target words under different learning contexts (monolingual, mixed-language). The results showed that, in the monolingual context, in the learning phase, the participants’ fixation time on the target was significantly higher than that in the mixed-language context (*t*(6) = 12.846, *p* < 0.001, *Cohen d* = 4.142). In the testing phase, in the monolingual context, the participants’ fixation time on the target was significantly higher than that in the mixed-language context (*t*(6) = 5.992, *p* = 0.001, *Cohen d* = 3.107) (Figure 3).

### 3.3. Combined Analysis of Experiments 1 and 2

A 2 (group: Chinese–English group, Chinese–Japanese group) × 2 (learning context: monolingual, mixed-language) analysis of variance was performed on the fixation time of the target words in Experiments 1 and 2, with “group” as the between-subject variable and “learning context” as the within-subject variable. Multiple comparisons between the conditions were performed using Bonferroni corrections.

A combined analysis of the fixation time in the learning phase was performed. The results of the analysis of variance showed that the main effect of the learning context was significant (*F*(1, 18) = 282.488, *p* < 0.001, *η^2^_p_* = 0.940), and the multiple comparison results showed that the fixation time of the target in the monolingual context was significantly higher than that in the mixed-language context (*p* < 0.001). The main effect of the group was not significant (*F*(1, 18) = 0.117, *p* = 0.736, *η^2^_p_* = 0.006). 

**Figure 4:**
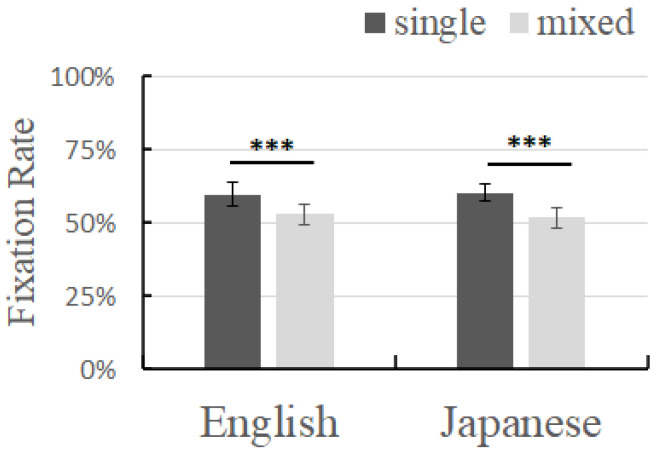
Fixation time of English and Japanese in different learning contexts in the learning phase; error bar represents the standard deviation. *** *p* < 0.001.

The results showed that the interaction effect between the learning context and the group was significant (*F*(1, 18) = 4.871, *p* = 0.041, *η^2^_p_* = 0.213), and the multiple comparison results showed that the fixation time of the target in the monolingual context was significantly higher than that in the mixed-language context in both the Chinese–English group and the Chinese–Japanese group (*p* < 0.001; *p* < 0.001) (Figure 4), and the difference in the fixation time of the target between the two contexts in the Chinese–Japanese group (521.940) was higher than that in the Chinese–English group (400.776). Further, a Bayesian paired-sample *t*-test was performed, with H0 indicating that there was no difference in the fixation time between the monolingual and mixed-language contexts. The results showed that, in the Chinese–English group, the fixation time of the target in the monolingual context was higher than that in the mixed-language context with *BF*_10_ = 2659.967, and, in the Chinese–Japanese group, the fixation time of the target in the monolingual context was higher than that in the mixed-language context, with *BF*_10_ = 469,029.993. According to the two Bayesian factors, the *BF*_10_ of the Chinese–Japanese group was 176.329 times that of the Chinese–English group, indicating that there was a very high probability of supporting the idea that the difference in the fixation time of the target between the two contexts in the Chinese–Japanese group was higher than that in the Chinese–English group.

The analysis of variance of the testing node showed that the main effect of the learning context was significant (*F*(1, 18) = 86.577, *p* < 0.001, *η^2^_p_* = 0.828), and the multiple comparison results showed that the fixation time of the target in the monolingual context was significantly higher than that in the mixed-language context (*p* < 0.001). The main effect of the group was significant (*F*(1, 18) = 5.688, *p* = 0.028, *η^2^_p_* = 0.240), and the multiple comparison results showed that the fixation time of the target in the Chinese–English group was significantly higher than that in the Chinese–Japanese group (*p* < 0.001).

The interaction effect between the learning context and the group was not significant (*F*(1, 18) = 0.127, *p* = 0.725, *η^2^_p_* = 0.007).

## 4. Discussion

In this study, we used eye-tracking technology to investigate the process of bilingual children learning new words in different mixed-language environments. The experimental purpose of this study was to explore the effect of a mixed-language environment on the vocabulary learning ability of bilingual children and compare the performance of vocabulary learning abilities in mixed-language environments composed of different language distance combinations (Chinese–English group and Chinese–Japanese group). We found that children can stably master new words, but the mixed-language environment inhibits children’s vocabulary learning. Notably, the consistency effect in the Chinese–Japanese mixed-language environment was stronger than that in the Chinese–English mixed-language environment.

First, in this experiment, the researchers strictly screened the participants to eliminate possible factors that might have interfered with the experimental results. For example, we conducted experiments on two groups of 4-year-old bilingual children: Chinese–English (Experiment 1) and Chinese–Japanese (Experiment 2). The participants chose children who had the same second-language learning time to ensure that they had basically the same English and Japanese abilities. These children learned the second language in a full-time kindergarten, which ensured that the participants avoided the influence of family and social background. And, the participants did not suffer from any language disorders or neurological diseases. In addition, during this experiment, the researchers strictly controlled the experimental conditions (such as sentence mixing form and language mixing degree) and test materials (such as the syllables of the created new words) for each participant to minimize the interference of confounding factors on the experimental results.

### 4.1. The Inhibitory Effect of Mixed-Language Environments

Our study showed that the main effect of learning context was significant, that is, the target fixation time in the monolingual environment was significantly higher than that in the mixed-language environment. According to the results of our analysis of variance and multiple comparisons, we found that the efficiency of learning new words in the mixed-language environment was lower than that in the monolingual environment, and this conclusion applied to both Chinese–English and Chinese–Japanese bilingual children. This indicates that the monolingual environment can better encode the connection between the new word and its referent, while the mixed-language environment interferes with the encoding of this connection.

The effect of mixed-language environments on children’s vocabulary acquisition has been a topic fraught with controversy. Both positive and negative effects have been found, and the present study provides more evidence for negative effects. Negative effects have been identified as having two main sources, the first being that mixed-language environments impose a higher cognitive load, and the second being that mixed-language environments reduce vocabulary in a variety of languages in everyday exposure. In the present study, the subjects learnt the vocabulary of one language in both monolingual and mixed-language environments, and the creation of mixed-language environments did not significantly reduce the vocabulary of one language, so the source of the negative effect found in the present study should be mainly traced to the cognitive load imposed by mixed-language environments.

In the mixed-language setting in this study, the target words and specific subjects that the children needed to learn were in their native language, but a familiar word was replaced with their second language. In the monolingual environment, the entire sentence was in the children’s mother tongue.

Since the second language occurred in the mixed-language environment during the learning phase, the subjects needed to deal with the interference of the second language during their learning of the first-language vocabulary. The mixed-language environment imposed a greater cognitive load, which reduced the children’s performance during the learning phase.

In addition, during the learning process, second-language familiar words and first-language target words are closely linked, and children learn first-language target words through the positional relationship between first-language target words and second-language familiar ones. This leads to the fact that, in the process of learning a first-language target word, children will inevitably establish a connection between the first-language target word and the second-language familiar one, in addition to establishing a connection between the first-language target word and the object.

According to models of the mental lexicon, the representations of the same concept in two languages are not completely isolated, but there are common representations [49,50]. On the one hand, a greater cognitive load at the learning stage may reduce the connection relation between first-language target words and objects, and, on the other hand, the connection between first-language target words and second-language familiar words may bring about interferences in the extraction process. The combined effect of these two aspects may have been responsible for the worse performance observed in the mixed-language environment during the testing phase of our study.

But, the negative effects of language mixing found in this study do not mean that language mixing is all negative for children. The duration of this study was short compared to a true mixed-language environment, and a short period of inhibition does not necessarily mean a long period of inhibition. In true second-language teaching, language switching is considered to be an effective and reliable method [51]. Also, the cognitive load of mixed-language environments does not imply damage to children’s cognitive abilities. If children are exposed to such high cognitive loads for a long period of time, their executive functions may also be exercised, which may have a beneficial effect on the improvement of said executive functions [52,53,54].

### 4.2. Moderating Effects of Language Distance on Inhibition

The joint analyses revealed an inhibitory effect of mixed contexts on vocabulary learning during the learning phase for both Chinese–English bilinguals and Chinese–Japanese bilinguals. It is worth noting that Chinese and Japanese bilinguals’ vocabulary learning was more inhibited.

Cui’s study used a database to calculate the linguistic distance between Chinese and many other languages, where the distance between Chinese and Japanese was 98.4, and the distance between Chinese and English was 102.4 [37], which means that the distance between Japanese and Chinese is closer than the distance between English and Chinese. The LDND indicator used in Cui’s study reflects the similarity in pronunciation between different languages, and the more similar the pronunciation, the closer the linguistic distance. In our study, although only a few vocabulary terms were used, the same situation existed, in that that the Chinese–English distance was greater than the Chinese–Japanese distance. Specifically, the English familiar words in Experiment 1 were monosyllabic words (pig, cat, dog), but the Japanese familiar words in Experiment 2 were disyllabic words (puta, neko, inu), and the monolingual target words in both experiments were disyllabic words. So, in Experiment 1, the learning of bisyllabic target words was interfered with by monosyllabic words, but, in Experiment 2, the interference came from bisyllabic words. Compared to the situation where target word was a disyllabic word and the familiar word was a monosyllabic word, the interference from both the target word and the familiar word being disyllabic words was greater. The subjects had to cope with interference from the same number of syllables in addition to the interference from two-syllable words.

Children need more cognitive resources to deal with the conflict caused by language similarity, but children lack the ability to resolve this conflict, resulting in more inhibition in the Chinese–Japanese group in our study. This also indicates that the ability of bilingual children to learn new words varies in different mixed-language environments with different language distances, that is, the closer the language distance, the greater the cognitive inhibition of vocabulary learning, and the farther the language distance, the smaller the cognitive inhibition of vocabulary learning.

### 4.3. Research Limitations

This study has some limitations. This study set up multiple recruitment criteria in order to better control extraneous variables, which led to a lower ecological validity of the results of this study. This study focused on native Chinese speakers whose second language was used less in daily life, so the results are difficult to generalize to groups who are non-Chinese speakers and whose second language is used more in daily life. In addition, again due to the recruitment criteria, the overall number of subjects in this study was relatively small, which somewhat weakens the reliability of the results.

## 5. Conclusions

For four-year-old Chinese–English and Chinese–Japanese bilinguals, vocabulary learning was worse in the mixed-language environment than in the monolingual environment. This inhibitory effect appeared at both the learning stage and the testing stage. This could be the result of the higher cognitive load in the mixed-language environment during the learning phase. In addition, this inhibition effect was moderated by the linguistic distance between the target languages in the monolingual and bilingual groups, with a closer linguistic distance enhancing this inhibition.

In future research, we can consider extending our experimental design, such as by using other tasks to explore the ability of language control and differentiation. We could also expand and improve the language materials, such as by using correct grammar to create new sentences as the experimental materials. At the same time, we could use other techniques to increase our understanding of the language processing process, such as by using brain imaging technology to explore the neural mechanisms of children in different mixed-language environments. Through these studies, we could gain a deeper understanding of the impact of different mixed-language environments on children’s language processing abilities and provide a more scientific theoretical and practical basis for language development and education in multilingual children. This could also provide a reference for the practice of language learning and child language development. For example, educators could consider providing children with a learning environment closer to being a single-language environment to help them learn new words better.

## Figures and Tables

**Table 1:** Fixation time (M ± SD) of different learning contexts in different phases.

	Single	Mixed
Learning phase	59.6% ± 4.1%	52.9% ± 3.0%
Test phase	66.8% ± 4.8%	56.3% ± 2.9%

**Table 2:** Fixation time (M ± SD) of different learning contexts in different phases.

	Single	Mixed
Learning phase	60.3% ± 3.7%	51.6% ± 3.5%
Test phase	64.1% ± 3.8%	52.7% ± 5.5%

## Data Availability

The data that support the findings of this study are available upon request from the corresponding author. The data are not publicly available due to privacy restrictions.
